# Traumatic brain injury among female offenders in a prison population: results of the FleuryTBI study

**DOI:** 10.1002/brb3.535

**Published:** 2016-10-21

**Authors:** Eric Durand, Laurence Watier, Anne Lécu, Michel Fix, Jean‐Jacques Weiss, Mathilde Chevignard, Pascale Pradat‐Diehl

**Affiliations:** ^1^ CNRS UMR 7371INSERM UMR S 1146Laboratoire d'Imagerie Biomédicale (LIB)UPMC Univ Paris 06Sorbonne UniversitésParisFrance; ^2^ Service de MPRFondation Sainte MarieParisFrance; ^3^ U 657InsermParisFrance; ^4^ Institut PasteurPhEMIParisFrance; ^5^ Faculté de Médecine Paris Ile de France OuestEA 4499Université Versailles Saint QuentinVersailles Saint QuentinFrance; ^6^ UCSA des maisons d'arrêt de Fleury‐MérogisSainte Geneviève des BoisFrance; ^7^ Centre Ressources francilien du traumatisme crânienParisFrance; ^8^ Service de Rééducation des pathologies neurologiques acquises de l'enfantHôpitaux de Saint MauriceSaint MauriceFrance; ^9^ Département de Médecine physique et de réadaptationAPHP Hôpitaux Universitaires Pitié‐Salpêtrière‐Charles FoixHôpital de la Pitié‐SalpêtrièreParisFrance

**Keywords:** female prisoners, head injury, inmates, neuropsychology, traumatic brain injury

## Abstract

**Aim:**

The study was designed to estimate the prevalence of traumatic brain injury (TBI) in a French prison population of female offenders, study the variables known to be associated with TBI, and compare our results with those obtained among male offenders as described in a previous paper.

**Participants:**

All female offenders (adults and juveniles) consecutively admitted to Fleury‐Mérogis prison over a 3‐month period were included in the study.

**Method:**

During the admission procedure, female offenders were interviewed by healthcare staff using a self‐reported questionnaire.

**Results:**

In all, 100 female offenders were included. The rate of self‐reported TBI was high, with a prevalence of 21%. The first cause of TBI was violence related (35%) and a majority of female offenders with a history of TBI reported having sustained more than one TBI. When compared with those who did not report a TBI, epilepsy and use of alcohol were higher among female offenders with a history of TBI. Perceived health was significantly worse for women who reported a TBI.

**Conclusions:**

This study findings provide additional evidence that TBI among offender populations is serious and that specific actions need to be developed and implemented in correctional settings such as screening for TBI upon arrival.

## 1 Introduction

Traumatic brain injury (TBI), which is one of the major causes of disability among working age adults (Fleminger & Ponsford, 2005) and represents a serious public health concern (Thurman, Alverson, Dunn, Guerrero, & Sniezek, 1999), has been identified in several studies as being strongly associated with offending (Farrer & Hedges, 2011; Shiroma, Ferguson, & Pickelsimer, 2010; Williams et al., 2010). Therefore, TBI should be part of the concerns when addressing the healthcare needs of the prison population.

A TBI is caused by a bump, blow, or jolt to the head or a penetrating head injury that disrupts the normal function of the brain (Centers for Disease Control and Prevention (CDC), National Center for Injury Prevention and Control, 2003). The most common causes of TBI are falls and road traffic accidents (RTA) (Peeters et al., 2015). Violence‐related mechanisms are less frequent and represent around 10% of all TBI in the general population (Langlois, Rutland‐Brown, & Wald, 2006).

TBI can result in lifelong cognitive, behavioral, and emotional consequences (Langlois et al., 2006). Cognitive deficits can be dominated by executive, memory, and attention dysfunction in adults (Mazaux et al., 1997). Behavioral disorders may cause social integration difficulties and may sometimes be associated with mood disorders (Baguley, Cooper, & Felmingham, 2006; Cattelani, Roberti, & Lombardi, 2008). There are particular issues regarding TBI occurring during childhood, as injury to the developing brain can alter the subsequent development of cognitive and behavioral functioning. Disorders of basic processes such as working memory and inhibition, and impairments of more complex processes such as decision making have been described following childhood TBI (Levin & Hanten, 2005). Other domains such as motivation, self‐regulation, and social cognition can also be affected (Levin & Hanten, 2005).

Criminality has also been reported as a possible outcome following TBI (Fazel, Lichtenstein, Grann, & Långström, 2011; Lukkainen, Riala, Laukkanen, Hakko, & Räsänen, 2012; Vaughn, Salas‐Wright, DeLisi, & Perron, 2014). In the Northern Finland Birth Cohort study, TBI was significantly related to later criminality in the male cohort members (Timonen et al., 2002). In a recent study, young, single, less educated men were significantly more likely to be arrested after a TBI (Elbogen et al., 2015).

Two recent meta‐analyses of studies in English‐speaking countries found the prevalence of a history of reported TBI among prisoners to be 60.25% and 41.2%, respectively (Farrer & Hedges, 2011; Shiroma et al., 2010). A significant difference was also found in the frequency estimate of TBI in the general population and the pooled estimate from the prison population (Farrer & Hedges, 2011). In another study, the prevalence of a history of TBI was 60.7% among a male population of 196 inmates (Williams et al., 2010). Several studies have highlighted the fact that prisoners with a history of one or more TBIs have significantly more health problems and significantly higher recourse to hospital care, use more alcohol or cannabis, and suffer significantly more psychiatric disorders (Barnfield & Leathem, 1998a, 1998b; Gunter, Philibert, & Hollenbeck, 2009; Schofield et al., 2006a, 2006b; Walker, Hiller, Staton, & Leukefeld, 2003; Walker, Staton, & Leukefeld, 2001). A previous study estimated the prevalence of self‐reported TBI in a representative population of male prisoners to be 32% in France (Durand et al., 2016).

Interestingly, the vast majority of published studies have included only male offender populations and little is known about female offenders. This “lack of interest” could be explained by the small proportion of female prisoners, usually less than 5% of the prison population in most European countries. However, the number of female prisoners has increased rapidly in some countries (e.g. in the United States, with a 646% increase between 1980 and 2010). In France, there was a 125% increase between 1980 and 2010. Only two studies on the prevalence of TBI among female offenders used an exclusively female population (Brewer‐Smyth & Burgess, 2008; Brewer‐Smyth, Burgess, & Shults, 2004). In the few studies which included men and more than 10 women, the prevalence of TBI among female offenders ranged between 9.5% and 72% (Brewer‐Smyth & Burgess, 2008; Brewer‐Smyth et al., 2004; Colantonio, Stamenova, Abramowitz, Clarke, & Christensen, 2007; Colantonio et al., 2014; Ferguson, Pickelsimer, Corrigan, Bogner, & Wald, 2012; Hawley & Maden, 2003; Hux, Bond, Skinner, Belau, & Sanger, 1998; Perron & Howard, 2008). These prevalences can be considered much higher that one would expect if the usual gender ratio were taken into account (the rate of TBI among men is twice the rate among women).

The aims of this study are threefold, the object being to answer the following questions: (1) What is the prevalence of self‐reported TBI among female offenders in France?; (2) What covariables are associated with a self‐reported TBI among female offenders?; and (3) What are the differences between female and male offenders who report having sustained a TBI?

The choice of a self‐reported questionnaire was made with reference to previous studies which showed the validity of such a method (Schofield, Butler, Hollis, & d'Este, 2011).

## 2 Methods

### 2.1 Setting

This study was conducted at Fleury‐Mérogis Prison which is located in the southern suburbs of Paris. This prison comprises a large men's jail, a women's jail, and a jail for male juveniles. According to the results of a national survey looking at the health status of all incomers in France, the health status of incomers in Fleury‐Mérogis can be considered as representative of that of the offender population in France (Mouquet, Dumont, & Bonnevie, 1999). The women's jail is designed to receive adult and juvenile females. In this study, the term juvenile refers to prisoners aged between 13 and 17, as in France the minimum age for imprisonment is 13. Also, the proportion of women is larger among the offender population of Fleury‐Mérogis than among the French prison population as a whole (6% vs. 3.5%). This difference can be explained by the fact that there are only a few prisons that can take female prisoners in the Paris area and Fleury‐Mérogis prison takes both women who have been sentenced and awaiting trial.

### 2.2 Population

All female offenders (adults and juveniles) admitted to Fleury‐Mérogis prison during a 3‐month period (11/01/2012 to 01/31/2013) were included in this study. During the routine admission procedure they were interviewed by healthcare staff (nurses and physicians), who had been trained earlier as to the aims of the study and the completion of a self‐report questionnaire.

### 2.3 Ethical concerns

Ethical approval for this study was provided by the University Paris VI (Pierre et Marie Curie University) and by the Consultative Committee for Treatment of Health Research Information which stated that written consent was not needed. Information about the study was given by a prison nurse or a prison doctor and informed consent for participation was given orally. Prisoners could refuse to participate without there being any consequences. Whenever possible, the reason for an interview refusal, or for noncompletion, was recorded.

### 2.4 Material

The questionnaire was administered during the systematic, semi‐structured interview carried out upon admission to the prison. It included demographic information, education level, and current work occupation, divided into five categories: worker (e.g., factory worker, carpenter), employee (e.g. office work), executive (e.g., executive, manager), unknown, or unemployed. It also included information about perceived health, history of TBI, history of epilepsy, any psychiatric and neurological follow‐up, questions about the use (daily, regularly, etc.) of alcohol, cannabis, or other psychoactive drugs. For alcohol use, two binary variables were defined: “Alcohol use” when offenders declared alcohol use during the last 30 days, as opposed to no use during the last 30 days, and “regular alcohol use” when offenders declared regular or daily alcohol use during the last 30 days, as opposed to no use or occasional use during the last 30 days. The same binary variables were defined for cannabis. Due to sample sizes, daily use could not be considered separately. Information about offending history was also recorded: number of previous imprisonments (in addition to the present one), total length of custody in the last 5 years, remand, or convicted status. In France, remand prisoners are awaiting trial, which means they have been accused of a crime but have not yet been proven guilty.

The first question about the incomer's history of TBI was translated from a questionnaire created by Williams et al. (2010): “Have you ever had an injury to the head that caused you to be knocked out and/or dazed and confused e.g. from a fall, blow to the head (including boxing or fighting) or road accident?” Whenever a TBI was reported, related information about the TBI was recorded: circumstance, age at first and last TBI (when appropriate), loss of consciousness (LOC), hospitalization, coma, and etc. As it was a declarative study, the Glasgow Coma Scale score could not be used and participants were asked “if they had been in a coma as a result of their TBI”. When the answer was “yes” a coma was recorded. TBI was considered as severe if there was a self‐reported coma, and as moderate if there was a self‐reported hospitalization without a coma. All the other self‐reported TBIs were considered as mild TBI. Prisoners were also asked if they had benefited from any specialized follow‐up after their TBI.

### 2.5 Statistical analyses

Firstly, the study population was described, distinguishing between juveniles (<18 years) and adults (≥18 years) females. Frequency distribution [*n* (%)], mean, and standard deviation (*SD*) were computed according to categorical and continuous variables. Regarding the nonnegligible number of missing data for some variables, percentages included missing value frequencies.

Comparisons were carried out between women who reported one or several TBI (cases) and those who did not (controls). Comparisons were also performed between female and male offenders who reported a TBI (Durand et al., 2016). Statistical analyses were performed using an exact Fisher test for categorical data and a nonparametric Wilcoxon signed‐rank test for quantitative data.

## 3 Results

### 3.1 Description of the prisoner population

A total of 100 women (88 adults and 12 juveniles) were admitted to Fleury‐Mérogis during the study period (Table**** 1) and all the questionnaires were answered. In the adult population, the mean age was 32.4 years. The education level was low with only 46.6% having completed more than 10 years of education. A large proportion (23.9%) was unemployed and the proportion of workers and employees was nearly a third of the total population (29.6%). They had had their first contact with the custodial system quite late (mean age 28.5 years). They had been in prison slightly less than twice and had spent less than 10 months in prison over the past 5 years. Nearly half (46.6%) were convicted. The mean age of juveniles was 15.5 years and only one had had more than 10 years of education.

**Table 1:** Characteristics^a^ of the study population for women (*N *=* *100)

	<18 years	≥18 years
	*N *=* *12	*N *=* *88
Age, mean (*SD*), years	15.5 (0.90)	32.4 (10.56)
>10 years education, *n* (%)	1	41 (46.6)
Occupation^b^, *n* (%)		
Worker	0	7 (8)
Employee	0	19 (21.6)
Executive	0	3 (3.4)
Unknown	2	9 (10.2)
Unemployed	1	21 (23.9)
Convict status^c^, *n* (%)	4	41 (46.6)
Age at first imprisonment^d^, mean (*SD*), yrs	15.25 (0.89)	28.5 (9.31)
Number of additional imprisonments^e^	1	1.6 (1.04)

a Because the samples were small, percentages were not computed for juvenile females. For adult females, they were computed with missing values taken into consideration. A note indicates when >15% of the data in a group were missing.

b 75% of missing data for juvenile females and >30% for adult females.

c 25% of missing data for juvenile females and >20% for adult females.

d >30% of missing data for juvenile females and >20% of missing data for adult females.

e 50% of missing data for juvenile females and 25% of missing data for adult females.

### 3.2 Description of the TBI population

The prevalence of a self‐reported history of TBI was 22.7% for adult females (*n *=* *20) and one female juvenile among 12 declared a history of TBI. Violence‐related outcomes (7/21) and road traffic accidents—RTA—(7/21) were the major causes of TBI (Table**** 2).

**Table 2:** Description^a^ of traumatic brain injury characteristics for female juveniles and adults

	<18 years	≥18 years
	*n*/*N* b	*n*/*N* b
Causes		
Road traffic accident	1/1	6/20
Sports accident	0	2/20
Fall	0	2/20
Violence related	0	7/20
Combination of causes	0	0
Other	0	2/20
Follow‐up after TBI	1/1	5/20
LOC	1/1	9/20
Coma	0	5/20
Hospitalization for TBI	1/1	11/20
More than one TBI	1/1	10/20
TBI after first imprisonment	0	5/20

TBI, traumatic brain injury; LOC, loss of consciousness excluding coma.

a Because the samples were small, percentages were not computed for this table.

b Number of cases/total number of nonmissing responses.

A large majority of the population with a history of TBI had no subsequent follow‐up related to their TBI (15/21 women). Of 21 women who reported a history of TBI, 10 reported a LOC (less than 24 hr), 5 a coma, and 12 a hospitalization. In other words, 5 women sustained a severe TBI (coma), 7 a moderate TBI (LOC and hospitalization), and 9 a mild TBI (LOC or not without hospitalization). Eleven women reported more than one TBI (11/21). In 5 cases, the first TBI occurred after the first imprisonment.

### 3.3 Medical and recreational drug use history

The prevalence of self‐reported epilepsy was 6.8% (*n *=* *6) for adult females (Table**** 3). A history of psychiatric care or follow‐up was reported by 11.4% of women. However, a notable proportion of women received anxiolytic treatment (23.9%), and 13.6% were receiving antidepressant treatment. Adult females used more alcohol than cannabis (21.6% vs. 12.5%). Daily or regular use of alcohol and cannabis was reported in 9.1% and 8.8% of cases, respectively.

**Table 3:** Medical history, drug, and alcohol use^a^ of the study population for females juveniles and adults^b^ (*N *=* *100)

	<18 years	≥18 years
	*N *=* *12	*N *=* *88
History of TBI	1 (8.3)	20 (22.7)
Epilepsy	0	6 (6.8)
Psychiatric care	0	10 (11.4)
Anxiolytic treatment^c^	0	21 (23.9)
Antidepressant treatment^d^	0	12 (13.6)
Alcohol use	0	19 (21.6)
Regular or daily use	0	8 (9.1)
Cannabis use^e^	1 (12.5)	11 (12.5)
Regular or daily use^e^	0	6 (8.8)

TBI, traumatic brain injury.

a Alcohol and cannabis use was evaluated for the preceding 30 days.

b Because the samples were small, percentages were not computed for juvenile females. For adult females, they were computed taking missing values into consideration, a note indicates when >15% of the data in a group were missing.

c 25% of missing data for juvenile females.

d 25% of missing data for juvenile females.

e 30% of missing data for juvenile females and >23% for adult females.

### 3.4 Comparisons between cases and controls

Cases were significantly older than controls (35 vs. 29; *p* = .01) (Table 4). No differences were found regarding the age of first contact with the prison (30.4 vs. 26.2; *p* = .21), the number of times in custody (2 vs. 1.4; *p* = .41), and the total time spent in jail during the preceding 5 years (10 month vs. 7.3; *p* = .38). Missing data for those two later variables were > 25%. Perceived health was significantly worse for cases than for controls (*p* < .0001). Use of alcohol and regular use of alcohol were significantly more frequent for cases than for controls (38.1% vs. 15%; *p* = .03 and 23.8% vs. 3.9%; *p* = .01, respectively). No differences were found regarding cannabis use, psychiatric follow‐up, and psychotropic drugs use.

**Table 4:** Comparison^a^ between cases and controls (*N *=* *99^b^)

	Cases	Controls	*p* c
	*N *=* *21	*N *=* *78	
Age, mean (*SD*), years	35.1 (10.71)	29.0 (11.26)	0.01
Perceived state of health, *n* (%)			
Very bad to average	14 (67)	17 (23)	<0.0001
Good to very good	7 (33)	57 (77)	
Epilepsy, *n* (%)	4 (19)	2 (2)	0.01
Psychiatric follow‐up, *n* (%)	2 (10)	8 (11)	1.00
Use of alcohol, *n* (%)	8 (38.1)	11 (15)	0.03
Regular use^d^ of alcohol, *n* (%)	5 (23.8)	3 (3.9)	0.01
Use of cannabis^e^, *n* (%)	3 (15.8)	9 (15.8)	1.00
Regular use^d^^,^ e of cannabis, *n* (%)	2 (9.5)	4 (7)	0.63
Anxiolytic treatment, *n* (%)	7 (33)	14 (19.8)	0.24
Antidepressant treatment, *n* (%)	5 (25)	7 (10.6)	0.14
Age at first imprisonment^f^, mean(*SD*), years	30.5 (11.76)	26.3 (8.96)	0.21
Total time in jail^g^, mean (*SD*), months	10 (8.65)	7.3 (10.22)	0.38
Number of imprisonment^h^, mean (*SD*)	2 (1.5)	1.4 (0.76)	0.41

a Percentages were computed taking missing values into consideration. A note indicates when >15% of the data in a group were missing.

b Among controls, one female did not answer the question about history of TBI.

c *p*‐value corresponding to Exact Fisher test for categorical data or nonparametric Wilcoxon signed‐rank test for quantitative data.

d Regular or daily use.

e 21% of missing data.

f 21% of missing data.

g 75% of missing data.

h 27% of missing data.

### 3.5 Comparisons between adult women and men who reported a TBI

Violence‐related outcomes were the first cause of TBI among women and were found in the same proportion among men (30% vs. 26.8%) (Table**** 5). No differences were found regarding the number of prisoners who reported more than one TBI, or their age at first and last reported TBI. No differences were found either between women and men regarding a follow‐up after their TBI. Perceived health was significantly worse for women than for men (*p* = .008). Although the use of anxiolytic and antidepressant treatments was more frequent among women than among men (33% vs. 15%; *p* = .03 and 23% vs. 11.3%; *p* = .07, respectively), no differences were found regarding psychiatric care. There was no difference between women and men regarding the prevalence of epilepsy. The use of cannabis was significantly more frequent among men (*p* = .03). No differences were found between the two groups regarding use of alcohol, regular use of alcohol, and regular use of cannabis.

**Table 5:** Comparison^a^ between women and men who self‐reported a TBI

	Adult females (*N *=* *20)	Adult males (*N *=* *317)	*p* b
*Description of traumatic brain injury characteristics*			
Causes, *n* (%)			
Road traffic accident	6 (30)	85 (26.8)	.87
Sports accident	2 (10)	24 (7.6)	
Fall	2 (10)	30 (9.5)	
Violence related	7 (35)	83 (26.2)	
Combination of causes	0	25 (7.9)	
Other	2 (10)	25 (7.9)	
More than one TBI, *n* (%)	10 (50)	110 (34.7)	.14
Age at first TBI, mean (*SD*), yrs	20.7 (10.48)	18.5 (8.87)	.35
Age at last TBI^c^, mean (*SD*), yrs	24.9 (12.56)	21.1 (9.12)	.19
Follow‐up after TBI, *n* (%)	5 (25)	52 (16.4)	.55
LOC, *n* (%)	8 (40)	149 (47)	.64
Coma, *n* (%)	5 (25)	50 (15.8)	.18
Hospitalization for TBI^d^, *n* (%)	10 (50)	127 (40.1)	.19
*Medical history, drug and alcohol consumption n (%)*			
Perceived state of health			
Very bad to average	13 (65)	104 (32.8)	.008
Good to very good	7 (35)	199 (62.8)	
Epilepsy	4 (20)	37 (11.7)	.29
Psychiatric care	2 (10)	69 (21.8)	.27
Anxiolytic treatment	7 (35)	45 (14.2)	.03
Antidepressant treatment	5 (25)	34 (10.7)	.07
Use of alcohol	8 (40)	192 (60.6)	.10
Regular use^e^ of alcohol	5 (25)	102 (33.2)	.62
Use of cannabis	3 (15)	136 (42.9)	.03
Regular use^e^ of cannabis	2 (10)	80 (25.2)	.17

TBI, traumatic brain injury; LOC, loss of consciousness excluding coma.

a Percentages were computed with missing values taken into consideration. A note indicates when >15% of the data in a group were missing.

b *p*‐value corresponding to Exact Fisher test for categorical data or nonparametric Wilcoxon signed‐rank test for quantitative data.

c 21% of missing data.

d 17% of missing data.

e Regular or daily use.

## 4 Discussion

This study addresses the prevalence of self‐reported TBI in a population of female prisoners in a French prison. Secondary aims included studying covariables known to be associated with TBI. The main findings were that the rate of self‐reported history of TBI was high, with an overall prevalence of 21%, and violence was the most frequent causes of TBI among women. The majority of reported TBIs were moderate or severe (57%). Furthermore, the rate of epilepsy was high (6%). Among women who reported a TBI, perceived health was worse and alcohol and psychotropic drugs use was more frequent than among those who did not report a TBI.

This study is the first to report on the prevalence of TBI among females prisoners in France. Among studies looking at the prevalence of TBI among females offenders (Bogner & Corrigan, 2009; Brewer‐Smyth et al., 2004; Colantonio et al., 2007, 2014; Diamond, Harzke, Magaletta, Cummins, & Frankowski, 2007; Ferguson et al., 2012; Gunter et al., 2009; Hawley & Maden, 2003; Hux et al., 1998; Lewis, Pincus, Feldman, Jackson, & Bard, 1986; Perron & Howard, 2008; Sarapata, Herrmann, Johnson, & Aycock, 1998; Slaughter, Fann, & Ehde, 2003), the rates vary from 9.5% (Perron & Howard, 2008) to 100% (Lewis et al., 1986). A rate of 100% was found in a very small sample of two women. When pooling the studies looking at the prevalence of TBI among female offenders (*n *=* *914), the average prevalence was 44% (Table**** 6). When pooling the studies that included more than 50 women (meta‐analysis excluded), the average prevalence was 39%. The rate of 21% found in our study is thus lower than previous findings. There are several possible reasons for this result: definitions of TBI are not always the same and lead to differences in reported rates (e.g., some studies define TBI as any head injury, whereas some consider TBI only if there has been a LOC); the populations studied are not similar and the purposes of the studies differ (e.g., in Colantonio's study (Colantonio et al., 2007)), the aim was to evaluate the prevalence of TBI among a forensic psychiatry population).

**Table 6:** Papers reporting on the prevalence of TBI among female offenders

References	Country	Population	Number of women	Age range, years (mean)	Method used	Definition of TBI	Prevalence rate among women (with LOC)
Lewis et al. (1986)	United States	15	2	NR	Psychiatric and neurological evaluation, psychological evaluation	All head injuries	100% (NR)
Sarapata et al. (1998)	United States	61	7				NR (NR)
Hux et al. (1998)	United States	316	105	11–20 (15.3)	Questionnaire sent to the parents	Blow to the head	37% (NR)
Hawley and Maden (2003)	United States	113	10	NR	Interview and questionnaire	Head injury with/without LOC	70% (NR)
Slaughter et al. (2003)	United States	69	6	NR for women	Interview	Mild/moderate and severe TBI	83% (NR)
Brewer‐Smyth et al. (2004)	United States	113	113	18–58 (33.2)	Interview and physical examination	TBI with LOC/coma	42% (42%)
Colantonio et al. (2007)	Canada	394	64	? – (39.9)	Retrospective chart reviews	TBI with or without LOC/mechanism	10.9% (NR)
Diamond et al. (2007)	United States	225	118	20–58 (36)	TBIQ	According to CDC definition (M, M, S)	NR
Perron and Howard (2008)	United States	723	94	11–20	Interview	Head injury with LOC>20 mn	9.5%
Gunter et al. (2009)	United States	330	116	Not specified – (33.9)	Interview and self‐report	Concussion with or without LOC > 5 mn	NR (NR)
Bogner and Corrigan (2009)	United States	210	105	18–55	Ohio State UniversityTBI identification method^a^	According to CDC definition (for MILD TBI)	NR (NR)
Shiroma et al. (2010)	M‐A	4865	387	NR	Meta‐analysis	All TBI or TBI with LOC	69.9% (55.28%)
Farrer and Hedges (2011)	M‐A	5049	NR	NR	Meta‐analysis	Any TBI reported as such	NR for women
Ferguson et al. (2012)	United States	636	316	19–63 (35.8)	Interview **+ **Ohio State UniversityTBI identification method	TBI with alteration of consciousness/TBI with LOC	72%
Colantonio et al. (2014)	Canada	235	104	18–45 (NR)	Interview (cf. Slaughter 2003)	TBI/LOC (< or > 30 min)	37.3% (30%)
Durand et al. (2016)	France	1148	100	14–67 (30.3)	Questionnaire	Mild/moderate and severe TBI	21% (14%)
Total^b^			914				44%
Total^c^			896				39%

a Head Injury Interdisciplinary Special Interest Group of the American Congress of Rehabilitation Medicine.

b Excluding studies where the percentage of TBI has not been rated and meta‐analysis (M‐A).

c Studies with more than 50 women with known rate of TBI, excluding meta‐analysis.

The prevalence of 21% found in this study confirms previous findings published in Canada and the United States (Bogner & Corrigan, 2009; Brewer‐Smyth et al., 2004; Colantonio et al., 2007, 2014; Diamond et al., 2007; Ferguson et al., 2012; Gunter et al., 2009; Hawley & Maden, 2003; Hux et al., 1998; Lewis et al., 1986; Perron & Howard, 2008; Sarapata et al., 1998; Slaughter et al., 2003). As data about the prevalence of TBI are lacking for Europe, it is hazardous to compare our findings with the prevalence of a history of TBI in the general population in France (Tagliaferri, Compagnone, Korsic, Servadei, & Kraus, 2006). However, the prevalence of women living with sequelae of TBI has been recently estimated to be 0.28% in the general population in France (Jourdan et al., [Link]). It is also very unlikely that the prevalence of TBI would be around 20% among the population as a whole in France.

Overall, the survey population was young (a mean age of 32.4 for adults and 15.5 for juveniles) with low levels of education and employment. In France, female prisoners are a few years older than male prisoners (a mean age of 35 vs. 33.4). These characteristics are similar to those usually found in offender populations, but also among populations having sustained a TBI (Godin‐Blandeau, Verdot, & Develay, 2013; Jourdan et al., 2013a, 2013b; Mouquet, 2005; Mouquet et al., 1999; Watson, Stimpson, & Hostick, 2004). According to our findings, cases were significantly older than controls (*p* = .01). Further research is needed to explain this difference.

The findings of this study also indicate that the leading causes of TBI in women are violence‐related mechanisms (7/21), and RTA (7/21). In France, according to a study performed in 1986 among the general population of the Aquitaine Region, 60% of TBI occurred following RTA and 33% following falls (Tiret et al., 1990). More recently, in the Paris region, the main causes of severe TBI were RTAs (53% of patients) and falls (35%) (Jourdan et al., 2013a). In the same study, violence‐related mechanisms were responsible for 5.4% of severe TBI. Interestingly, in our study, among women who reported violence‐related TBI, a large majority (5/7) reported more than three TBIs. As there is little research allowing information on women to be compared, one could argue that they are at a higher risk of repeated TBI from domestic violence (Ferguson et al., 2012).

In our population and according to the definition used, moderate or severe TBI accounted for 57% of all declared TBIs which is far more important than the percentage usually found for the TBI population as a whole (10 to 25%). These results are also quite different from some studies which indicate that women are more likely to have a high prevalence of mild TBI (Diamond et al., 2007). On the other hand, a recent study highlighted that gender differences, if they exist regarding the severity of TBI, could be explained by gender‐related behavioral patterns and factors (e.g., partner violence) and a reduced likelihood of reporting mild TBI (O'Sullivan, Glorney, Sterr, Oddy, & Da Silva, 2015).

In this study, the rate of self‐reported epilepsy (6.8%) was much higher than in the population as a whole (0.5% in France) [67–68]. Epilepsy was also found to be much higher for cases (19%) than for controls (2%) (*p* = .01). It was similar to the prevalence found among male offenders, which was 6% (Durand et al., 2016). Our study also confirmed previous findings about the high prevalence of epilepsy among offenders in France (Mouquet, 2005; Mouquet et al., 1999). Until now, there have been no plausible explanations for the difference in the prevalence of epilepsy in the prison and general populations (Durand & de Beaurepaire, 2001). One could argue that moderate or severe TBI is sometimes responsible for post‐traumatic epilepsy. Therefore, the high prevalence of epilepsy in this population could be linked to the high prevalence of moderate to severe TBI (Ferguson et al., 2010). Epilepsy can also cause falls and therefore could be one reason for the high prevalence of TBI in this prison population. Another explanation could be that prisoners report epilepsy in order to obtain the medication they use as psychoactive drugs (e.g., benzodiazepines). However, this is not really plausible, as the proportion of prisoners using those psychoactive drugs has been relatively stable since 1998 (Mouquet et al., 1999). Interestingly, the prevalence of epilepsy has increased dramatically over the last 15 years, from 1.5% in 1998 to 6% in this study. One could also argue that healthcare staff members are now more aware of TBI and epilepsy than they were a few years ago. However, this is very unlikely because no training has been organized in those areas, either recently or at the time of the first studies on the health status of prisoners (Mouquet, 2005; Mouquet et al., 1999).

Perceived health was worse for women who reported a TBI than for those who did not. Perceived health was also worse for women than for men. That could be explained by the fact that women who reported a TBI accumulate multiple health problems, including regular use of alcohol and psychotropic drugs. Among female cases, use of alcohol and regular use of alcohol were significantly more frequent than among female controls (*p* = .01). Alcohol has been clearly identified as a factor responsible for TBI and a complicating factor for rehabilitation (Jourdan et al., 2013a; Walker et al., 2001). In the PariS‐TBI study, 16% of patients who sustained a severe TBI reported preinjury alcohol abuse (Jourdan et al., 2013a). Moreover, a history of alcohol abuse has been identified as having a significant effect on the decision to refer a patient to a rehabilitation unit or not (Jourdan et al., 2013a, 2013b).

In our sample, psychotropic drugs use was more frequent for women than for men. These findings are not surprising and are in accordance with other studies looking at the association between TBI and the use of psychotropic drugs (Walker et al., 2001). In any case, mental health issues are frequent following preceding TBI (Timonen et al., 2002), as well as among prisoners, the most frequently reported disorder being depression (Schofield et al., 2006a, 2006b; Slaughter et al., 2003; Walker et al., 2003).

The results of this study also highlight the question of the association between criminality and TBI among female offenders. Differences between women and men should be studied because this might lead to different management strategies for offenders (Styrke, Sojka, Björnstig, Bylund, & Stålnacke, 2013). One study recently addressed the differences between male and female offenders (Colantonio et al., 2014). In this study, more TBIs occurred before the first crime for women than for men and women with TBI experienced more physical and sexual abuse. In our study, the first TBI occurred before the first imprisonment in the majority of cases among women (76%) and even more frequently (86%) among men (Durand et al., 2016).

Even if the nature of the relationship between TBI and criminality needs further investigation, these findings could be of major interest for the management and rehabilitation of offenders. As some studies suggest that female offenders have often been abused physically and sexually (Brewer‐Smyth & Burgess, 2008; Brewer‐Smyth et al., 2004; Shiroma et al., 2010), they are at greater risk of sustaining a TBI. TBI itself may also be responsible for aggression and violent behaviors (Tateno, Jorge, & Robinson, 2003). In our sample, the leading cause of reported TBI was violence‐related mechanisms (7/21) and of those seven, five reported more than three TBIs. Multiple, mild TBIs can lead to cognitive and behavioral profiles similar to those seen following severe TBI (Diamond et al., 2007). Moreover, our findings indicate that 71% of female offenders who reported a TBI did not benefit from any kind of specialized follow‐up of any kind. As they can be associated with TBI, the focus should be on the early evaluation of aggressive and violent behaviors during the first weeks or months following a TBI. Rehabilitation should involve, whenever required, professionals from the health and social fields (Rosenbaum & Hoge, 1989). Whenever patients become involved with the criminal system, professionals from the justice field should also be involved because prisoners with a history of TBI may have more difficulties adapting to prison life (O'Sullivan et al., 2015). A specific gender approach should also be developed because female offenders with a TBI history can also be mothers, and aggression, abuse, and TBI must be prevented where their children are concerned. Previous studies by the French Ministry of Justice and Ministry of Health showed that the offender population in France had less access to care and were in a worse state of health than the general population (Chodorge, Nicolas, Colin, & Fuchs, 1993). Since, as far as we know, there has is no screening for TBI on arrival in prison in France, this could lead to further inequities in access to medical care.

### 4.1 Limitations

Our study is cross‐sectional and as data were collected by means of an interview (self‐report), the results should be interpreted with caution. As, however, the reliability of prisoners' responses is frequently challenged, it is interesting that a recent study involving 200 Australian prisoners highlighted the fact that inmates' responses to questionnaires corresponded closely to the reality (Schofield et al., 2011). These findings run counter to the conventional wisdom that responses by the criminal population are dishonest and therefore unusable.

One could also argue a recall bias, which is an error caused by differences in the accuracy or completeness of the recollections retrieved by participants regarding past events or experiences. Even if cases and controls were not of the same mean age (35 for cases, 29 among controls), findings about differences can be considered sound enough to be discussed because it is difficult to believe that anyone would not remember having sustained a TBI.

Neither of these limitations affects the primary aims of this study which were to establish the prevalence of TBI in a representative female population of prisoners in a French prison.

## 5 Conclusion

This study confirms the high prevalence of TBI among female prison inmates. Among cases, alcohol use was significantly higher and perceived health was significantly worse. No difference was found between women and men except for perceived health and cannabis use. Even if these results can be considered preliminary, they are consistent with previous studies using the same method. They also provide further evidence that, even though TBI has previously been identified as an issue for offender populations, there is a need to develop a specific policy for female offenders with a history of TBI. Indeed, further research is needed to better understand the relationship between TBI and violence which, in our study, is the first cause of TBI. The first steps would be to develop systematic screening for TBI upon arrival in prison, to build up knowledge about its prevalence and its causes, and to organize training for prosecutors, judges, and physicians working in prisons. Further studies should help in understanding and proving (or not) the possible association of TBI with criminality. Future steps should include carrying out a cohort study with the object of clarifying the possible links between these two issues.

## Conflicts of Interest

The authors of this manuscript declare there to be no conflict of interest.

## Funding Information

This study was funded by the “Fondation des Gueules Cassées” and has been supported by the “Centre Ressources du Traumatisme Crânien Ile de France” and Sorbonne Univ Paris 06, UMR 7371, UMR S 1146, Laboratoire d'Imagerie Biomédicale, F‐75005, Paris, France.
